# Supplementary material for: Effects of trust-based decision making in disrupted supply chains
Source: PLoS One. 2020 Feb 18;15(2):e0224761. doi: 10.1371/journal.pone.0224761 (PMC7028279; doi:10.1371/journal.pone.0224761)
Supplement: S1 Appendix — (PDF) [file pone.0224761.s001.pdf]

Robustness of Cost Changes Under *Short* Disruption

We tested the robustness of scenario 2 results around changes in agents costs if one healthcenter uses trustworthiness to order compared to when both split order equally. In order to do this, we run simulations with the same settings as scenario 2, but with normally-distributed patient demands. We ran simulations with a mean demand of 120 and 3 different standard deviation values, 5, 10, and 15. For each standard deviation we ran 50 different simulations.

For all agents (except *Equal HC*) and the supply chain as a whole we tested the hypothesis that mean of agent (supply chain)'s cost is smaller when both healthcenters split their order equally compared to when *Trust HC* uses trustworthiness to split its order. Thus we performed Welch's t-test to test the following hypothesis, using significance level of $\alpha = 0.05$:

$\boldsymbol{H_0}$: the mean of agent's (supply chain's) cost when both healthcenters split their order equally, is equal to the mean of agent's (supply chain's) cost when *Trust HC* uses trustworthiness to split its order.

$\boldsymbol{H_1}$: the mean of agent's (supply chain's) cost when both healthcenters split their order equally, is smaller than mean of agent's (supply chain's) cost when *Trust HC* uses trustworthiness to split its order.

As shown in Table 1, for all three levels of standard deviation, *Trust HC*'s *p-values* are smaller than $\alpha = 0.05$, so we reject the null hypothesis in favor of the alternative hypothesis.

Similarly for *Not-disrupted DS*, for all three standard deviation values *p-values* are smaller than the significance level of $\alpha = 0.05$, so we reject the null hypothesis in favor of the alternative hypothesis. As for *Disrupted DS* for a standard deviation of 5 and 10, we have the same conclusion and we reject the null hypothesis. However when the standard deviation is 15 we fail to reject the null hypothesis.

As for manufacturers, for all three standard deviation values *p-values* of both *Not-disrupted MN* and *Disrupted MN* are smaller than $\alpha = 0.05$, so we reject the null hypothesis in favor of the alternative hypothesis.

For the supply chain as a whole, the overall cost structure is more complex since it is the summation of costs across all echelons. Therefore, for standard deviation values of 10 and 15, we fail to reject the null hypothesis and there is no statistically significance difference between the cost of two scenarios at the significance level of $\alpha = 0.05$.

For *Equal HC* we perform Welch's t-test to test the following hypothesis:

$\boldsymbol{H_0}$: the mean of *Equal HC*'s cost when both healthcenters split their order equally is equal to the mean of *Equal HC*'s cost when *Trust HC* uses trustworthiness to split its order.

$\boldsymbol{H_1}$: the mean of *Equal HC*'s cost when *Trust HC* uses trustworthiness to split its order is smaller than the mean of *Equal HC*'s cost when both healthcenters split their order equally.

As shown in Table 1, for all three standard deviation values *p-values* are smaller that $\alpha = 0.05$, so we reject the null hypothesis in favor of the alternative hypothesis.

To summarize, while for lower variability of demand, and correspondingly smaller standard deviation values, results from scenario 2 are robust, when the demand variability increases it interacts with other features of the disrupted supply chain network, such as network structure, available decision calculations

Table 1. Results of Welch's t-test for different agents and supply chain as a whole under *Short* disruption (*p-values* are one-tailed *p-values*).

	Demand N(120, 5)		Demand N(120, 10)		Demand N(120, 15)	
	t-statistic	p-value	t-statistic	p-value	t-statistic	p-value
Trust HC	35.564	< 0.001	18.747	< 0.001	11.942	< 0.001
Equal HC	39.764	< 0.001	25.685	< 0.001	17.622	< 0.001
Not-disrupted DS	66.312	< 0.001	28.840	< 0.001	14.890	< 0.001
Disrupted DS	5.730	< 0.001	2.207	0.015	1.397	0.083
Not-disrupted MN	40.394	< 0.001	16.783	< 0.001	8.405	< 0.001
Disrupted MN	8.814	< 0.001	3.795	< 0.001	2.495	0.007
Supply Chain	3.602	< 0.001	-0.943	0.174	-1.484	0.070

Python 2.7 statistical functions package (scipy.stats) was used to calculate values in the table.

of agents and disruption profile. As a future step, we will study such interactions and the implications for a disrupted supply chain.

Robustness of Cost Changes Under *Long* Disruption

We tested the robustness of scenario 4 with $\delta = 0.5$ results around changes in agents costs if one healthcenter uses trustworthiness to order compared to when both split order equally. In order to do this, we run simulations with the same settings as scenario 4 with $\delta = 0.5$, but with normally-distributed patient demands. We ran simulations with a mean demand of 120 and 3 different standard deviation values, 5, 10, and 15. For each standard deviation we ran 50 different simulations.

For *Trust HC*, *Equal HC*, *Disrupted DS*, *Disrupted MN* and overall supply chain we perform Welch's t-test to test the following hypothesis:

$\boldsymbol{H_0}$: the mean of agent's (supply chain's) cost when both healthcenters split their order equally is equal to the mean ofagent's (supply chain's) cost when *Trust HC* uses trustworthiness to split its order.

$\boldsymbol{H_1}$: the mean of agent's (supply chain's) cost when *Trust HC* uses trustworthiness to split its order is smaller than the mean of agent's (supply chain's) cost when both healthcenters split their order equally.

As shown in Table 2, for all three levels of standard deviation, agents' and supply chain's *p-values* are smaller than $\alpha = 0.05$, so we reject the null hypothesis in favor of the alternative hypothesis.

For *Not-Disrupted DS* and *Not-Disrupted MN* we perform Welch's t-test to test the following hypothesis:

$\boldsymbol{H_0}$: the mean of agent's cost when both healthcenters split their order equally, is equal to the mean of agent's cost when *Trust HC* uses trustworthiness to split its order.

$\boldsymbol{H_1}$: the mean of agent's cost when both healthcenters split their order equally, is smaller than mean of agent's cost when *Trust HC* uses trustworthiness to split its order.

Similar to other agents' result, all *p-values* are smaller than $\alpha = 0.05$, so we reject the null hypothesis in favor of the alternative hypothesis.

In conclusion, results from changes in agents costs if one healthcenter uses trustworthiness to order compared to when both split order equally also holds when the demand is stochastic with low variability. As stated in the previous section one of our future research is introducing effect of demand variability in studying trust dynamics and mitigation of disruptions.

Table 2. Results of Welch's t-test for different agents and supply chain as a whole under *Long* disruption (*p-values* are one-tailed *p-values*).

	Demand N(120, 5)		Demand N(120, 10)		Demand N(120, 15)	
	t-statistic	p-value	t-statistic	p-value	t-statistic	p-value
Trust HC	101.470	< 0.001	51.585	< 0.001	33.477	< 0.001
Equal HC	157.712	< 0.001	79.307	< 0.001	51.648	< 0.001
Not-disrupted DS	116.252	< 0.001	60.723	< 0.001	33.494	< 0.001
Disrupted DS	147.129	< 0.001	71.740	< 0.001	46.763	< 0.001
Not-disrupted MN	64.046	< 0.001	30.468	< 0.001	16.231	< 0.001
Disrupted MN	141.877	< 0.001	69.832	< 0.001	45.680	< 0.001
Supply Chain	139.439	< 0.001	68.644	< 0.001	44.590	< 0.001

Python 2.7 statistical functions package (scipy.stats) was used to calculate values in the table.

Robustness of Cost Changes Under *Moderate* Disruption

We tested the robustness of scenario 5 with $\delta = 0.5$ results around changes in agents costs if one healthcenter uses trustworthiness to order compared to when both split order equally. In order to do this, we run simulations with the same settings as scenario 4 with $\delta = 0.5$, but with normally-distributed patient

demands. We ran simulations with a mean demand of 120 and 3 different standard deviation values, 5, 10, and 15. For each standard deviation we ran 50 different simulations.

For *Trust HC*, *Not-Disrupted DS* and *Not-Disrupted MN* we perform Welch's t-test to test the following hypothesis:

$\boldsymbol{H_0}$: the mean of agent's cost when both healthcenters split their order equally, is equal to the mean of agent's cost when *Trust HC* uses trustworthiness to split its order.

$\boldsymbol{H_1}$: the mean of agent's cost when both healthcenters split their order equally, is smaller than mean of agent's cost when *Trust HC* uses trustworthiness to split its order.

As shown in Table 2, for all three levels of standard deviation, *Trust HC*'s *p-values* are smaller than $\alpha = 0.05$, so we reject the null hypothesis in favor of the alternative hypothesis. However, for *Not-Disrupted DS* and *Not-Disrupted MN* we fail to reject the null hypothesis for standard deviation of 10.

For *Equal HC*, *Disrupted DS*, *Disrupted MN* and overall supply chain we perform Welch's t-test to test the following hypothesis:

$\boldsymbol{H_0}$: the mean of agent's (supply chain's) cost when both healthcenters split their order equally, is equal to the mean of agent's (supply chain's) cost when *Trust HC* uses trustworthiness to split its order.

$\boldsymbol{H_1}$: the mean of agent's (supply chain's) cost when *Trust HC* uses trustworthiness to split its order is smaller than the mean of agent's (supply chain's) cost when both healthcenters split their order equally.

Similar to *Not-Disrupted DS* and *Not-Disrupted MN* results, we fail to reject the null hypothesis for standard deviation of 10 for these agents and the overall supply chain.

In conclusion, we observed that demand variability interacts with supply chain profile parameters and this in turn alters the suggested course of action in face of disruption. In our future work we aim to further investigate the effcet of demand variability in trust dynamic between supplier and buyer.

Table 3. Results of Welch's t-test for different agents and supply chain as a whole under *Moderate* disruption (*p-values* are one-tailed *p-values*).

	Demand N(120, 5)		Demand N(120, 10)		Demand N(120, 15)	
	t-statistic	p-value	t-statistic	p-value	t-statistic	p-value
Trust HC	59.041	< 0.001	45.927	< 0.001	27.760	< 0.001
Equal HC	171.116	< 0.001	82.936	< 0.001	61.594	< 0.001
Not-disrupted DS	5.387	< 0.001	1.625	0.054	31.476	< 0.001
Disrupted DS	6.499	< 0.001	2.033	0.023	7.813	< 0.001
Not-disrupted MN	4.697	< 0.001	0.462	0.323	18.324	< 0.001
Disrupted MN	5.474	< 0.001	1.408	0.081	4.801	< 0.001
Supply Chain	5.530	< 0.001	0.657	0.256	4.975	< 0.001

Python 2.7 statistical functions package (scipy.stats) was used to calculate values in the table.
