# Supplementary material for: Effects of trust-based decision making in disrupted supply chains
Source: PLoS One. 2020 Feb 18;15(2):e0224761. doi: 10.1371/journal.pone.0224761 (PMC7028279; doi:10.1371/journal.pone.0224761)
Supplement: S1 Table — (PDF) [file pone.0224761.s002.pdf]

| **Agent** | **Change in Agent's Cost** |
|---|---:|
| *Trust HC* | 72% |
| *Equal HC* | -88% |
| *Not-Disrupted DS* | 15% |
| *Disrupted DS* | -337% |
| *Not-Disrupted MN* | 7% |
| *Disrupted MN* | -169% |
| *Overall Supply Chain* | -9% |
